# A validation of the Swedish version of the WORC index in the assessment of patients treated by surgery for subacromial disease including rotator cuff syndrome

**DOI:** 10.1186/s12891-016-1014-y

**Published:** 2016-04-14

**Authors:** Soheila Zhaeentan, Markus Legeby, Susanne Ahlström, André Stark, Björn Salomonsson

**Affiliations:** Karolinska Institute, Division of Clinical Sciences, Danderyds University Hospital, 182 88 Stockholm, Sweden; Karolinska Institute Danderyds University Hospital, Stockholm, Sweden; Division of Orthopedics, Karolinska Institute Danderyds University Hospital, Stockholm, Sweden; Division of Orthopaedics Danderyds University Hospital, Stockholm, Sweden

**Keywords:** Rotator Cuff, Shoulder, WORC, Quality of Life, Validity, Reliability, Responsiveness, Questionnaire

## Abstract

**Background:**

The Western Ontario Rotator Cuff Index (WORC) is a widely used instrument to measure quality of life in patients with subacromial pain or rotator cuff syndrome.

The purpose of this study was to evaluate the psychometric properties of the Swedish version of the WORC for assessment of subacromial disease including rotator cuff syndrome treated by surgery.

**Methods:**

A total of 65 patients were included in this study, mean age 60 years (range 36–82), 42 % women, all were candidates for surgery for subacromial pain conditions at two orthopedic units during 2004–2006 and 2011–2012. Calculations of the validity of Pearson’s correlation coefficient, floor and ceiling effects, reliability and responsiveness have formed the basis of assessment of the WORC index properties. WORC has been tested against Western Ontario Osteoarthritis of the Shoulder (WOOS), Oxford Shoulder Score and EQ-5D.

An additional 49 patients, mean age 64 years (range 36–74) 20 % of whom were women, were analyzed in a WORC test-retest with ICC and also correlated to Constant-Murley Score.

**Results:**

The validity analysis of WORC showed high correlations with both the specific and the generic health measurement instrument. The reliability calculations of the WORC resulted in ICC = 0.97 and Cronbach’s alpha = 0.97. Responsiveness was also excellent for WORC with Effect size = 1.35 and Standardized Response mean = 1.01. We found that the WORC showed a strong correlation with the WOOS (0.97) and the Constant-Murley Score (0.85). A good correlation was found with the Oxford Shoulder Score (0.74) and the EQ-5D (0.71).

**Conclusions:**

The Swedish version of WORC can be considered reliable, valid and responsive for use as an assessment of outcome and a health measurement instrument for patients treated by surgery for subacromial disease including rotator cuff syndrome.

## Background

The Western Ontario Rotator Cuff Index (WORC) is a tool for evaluating shoulder function, based on a subjective self-evaluation by the patient [1]. WORC is also an increasingly applied disease-specific outcome measure for rotator cuff (RC) conditions [2]. The rationale for using the WORC for evaluating Quality of Life (QoL) when assessing shoulder patients is well documented, and the WORC has become one of the most commonly used health instruments specific for rotator cuff conditions [3]. It can be used without a clinical examination and is answered in its entirety by the patient, and is thereby defined as a patient-administered questionnaire. Other examples of patient-administered questionnaires used for subacromial shoulder conditions are the Western Ontario Osteoarthritis of Shoulder Index (WOOS) which was developed for evaluation of osteoarthritis of the shoulder by Lo et al. [4] in Canada, but has later on been assessed for evaluation of subacromial pain [5], and the Oxford Shoulder Score (OSS) [6]. The Constant-Murley score (CS) is currently one of the most frequently used tools for evaluating shoulder function, but the CS requires objective measurements and has even been criticized for having low inter-rater reliability [7–9]. This makes the CS less appropriate when comparing outcomes between different shoulder-treatment centers. In contrast, the WORC has been constructed for use in multicenter studies and for use in post-operative follow-up [1].

The WORC was originally introduced and published by Kirkley et al. in 2003 [1]. It was developed as a response to the lack of well-constructed instruments for measuring QoL in patients with rotator cuff syndrome. The original version of the WORC was created in English, and the psychometric evaluation that was made can therefore be considered valid only in that language [10]. Since 2003, the WORC has been translated and psychometrically evaluated in at least nine languages [11–16].

The WORC comprises 21 items that address symptoms in five domains; physical symptoms - 6 items, sport/recreation - 4 items, work - 4 items, lifestyle - 4 items, and emotions - 3 items. Each item’s response is presented on a visual analogue scale of 0–100, where 0 represents the least amount of symptoms and 100 represents the worst symptoms. The results can be calculated for each separate domain, as well as providing a total score ranging from 0 (least symptoms) to 2100 (worst symptoms). The total score can be recalculated to represent a percentage of a healthy shoulder, with 100 % being the best score depicting a healthy shoulder. Recalculation is performed using the formula (2100 – “patient WORC score”/21).

The Minimal Clinically Important Change (MCIC) in WORC has been calculated to be 275 points, or 12.8 % if presented in the mode of WORC% [17].

This is in line with the developers of the WORC index own description of Minimally Important Difference (MID) 11.7 % [1].

An approved translation into Swedish of the WORC was used for the purpose of this study. The WORC was compared to a similar shoulder score for osteoarthritis, the Western Ontario Ostheoarthritis of the Shoulder index (WOOS) which has already been psychometrically evaluated by Klintberg et al. in 2012 in a Swedish version for patients with subacromial pain [5]. Klintberg et al. compared the WOOS with the Swedish version of the Shoulder Rating Questionnaire and found that the WOOS was valid, reliable, and responsive in evaluating patients with subacromial pain conditions. Our choice of questionnaires to test against WORC was based on the fact that OSS and WOOS were developed using modern techniques, they were translated into Swedish in 2005 according to recommended standards and they are well established [10]. The Constant-Murley score is the one that has been recommended by the European Society of Shoulder and Elbow Surgery (SECEC) for a very long time, and it is also still in common use in Sweden. The Euro-Qol generic health instrument version European Quality of Life- 5 Dimensions 3 L (EQ-5D) is the most commonly used generic questionnaire for assessment of quality of life in Sweden.

The purpose of this study was to assess the validity, reliability, and responsiveness of the Swedish version of the WORC score in the evaluation of subacromial pain in patients treated by surgery.

## Methods

The study sample included patients with subacromial pain, or a rotator cuff tear, treated surgically. The participants took part either as pre- and postoperative participants (group 1), or as test-retest participants (group 2). A sample size recommendation for validation studies indicates that approximately 50 patients would be required in this study.

### Group 1

The patients (group 1) were recruited from routine patients at one orthopedic department in 2004–2006 (47 patients) and at another orthopedic unit during 2011–2012 (18 patients). These patients were included in the study since they met the following criteria: 1: They were diagnosed with a subacromial disease such as impingement, biceps tendonitis or rotator cuff tears or a combination of these diseases. 2: They were candidates for surgical treatment. 3: They agreed to participate.

A total of 65 patients were included and they answered three different disease-specific questionnaires (WORC, WOOS, and OSS) and an additional EQ-5D, both pre- and postoperatively. The mean age of the patients was 60 years (range 36–82 years), and 27 (42 %) were women. Four patients failed to complete all the questionnaires.

### Group 2

Group 2 consisted of a total of 49 patients, who answered the WORC twice, in a test-retest manner. These 49 patients were retrieved from research material in a previously published retrospective study by Zhaeentan et al. of 73 patients treated using open rotator cuff surgery [18]. At the time of follow-up the patient symptoms were considered clinically stable, and met the following inclusion criteria:

1. Previous rotator cuff tear treated surgically 1–10 years earlier.
2. 18 years of age and above.
3. Able to read and understand spoken Swedish.

When these patients presented at the clinic for their follow-up during 2011–2012 they were asked to participate in a WORC test-retest. All of them had already completed a postoperative WORC questionnaire at minimum one year after the surgical treatment. None of the 49 participants who agreed to participate had experienced a change in symptoms between the tests, and this was considered to be a large enough sample size to achieve a reliable result in intraclass correlation (ICC) calculations. The time between test-retest was 36 to 367 (on average 108) days, the mean age of the participants was 64 years (range 36–74 years) and 10 (20 %) of them were women.

If a WORC or WOOS questionnaire had more than three answers missing, it was completely discarded (two questionnaires), 26 questionnaires had one or two answers missing and in these cases, answers missing were compensated by either imputation of a domain average or a total average [19]. All statistical calculations of the WORC and WOOS scores were made using the scores in the 0 (least symptoms) - 100 (worst symptoms) range. Due to diverse calculation of the scoring in different health measurement instruments, a correlation between scores could be negative in some cases. To avoid the confusion of negative figures, the absolute values have been used.

The number of questionnaires analyzed varied due to the exclusions of incomplete questionnaires, and was between 126 and 129 depending on which two scores were correlated. Since every patient in study group 1 produced both pre- and postoperative results, and the correlation was calculated score by score, every patient contributed twice in the same correlation calculation. This widened the range of measures available for correlations with both pre- and post-operative measurements.

For the calculation of the postoperative satisfaction level (SL) we only had results from some of the participants in study group 1; resulting in a smaller sample size, 45 (of 65) patients. The SL was also compared to the difference in pre- and postoperative WORC-scores to determine whether patients with a larger difference between the pre- and postoperative WORC were also more satisfied with their treatment.

#### Statistics

The following methodology was applied in the individual statistical tests:

The co-variance of the instruments was calculated using the Pearson’s correlation coefficient (PCC) or the Spearman correlation coefficient (SCC). The SSC is a non-parametric alternative to the PCC.

The PCC was calculated using the pre- and postoperative material from group 1 for correlation assessment and was calculated individually for the WORC, WOOS, OSS, and EQ-5D.

The SCC was calculated for the correlation between SL and the WORC’s total score.

Furthermore, the PCC was calculated with respect to test and retest WORC scores. The correlation with the test-retest material could then be compared to the correlation calculated between WORC and WOOS scores.

### Content validity

Floor and ceiling effects were calculated pre- and postoperatively for patients in group 1. In the current study, 0–1 % (the final score percentage) was considered to be the lowest possible value and 99–100 % was considered to be the highest possible index value for the WORC and WOOS instruments (representing a possible measurement error of 1 mm on the VAS scale). The EQ-5D had a lowest possible value of -0.594 and a highest possible value of 1.0, which were thus considered to be floor and ceiling values, respectively. The OSS had a lowest possible value of 12 points (floor) and a highest possible value of 60 points (ceiling). The SL was not included in the content validity analysis as it had only one question with four Likert scale alternatives (1.very satisfied, 2. satisfied, 3. neither satisfied nor unsatisfied, 4. unsatisfied) leaving 50 % of the alternatives as either floor or ceiling results. Hence, the SL could not add any information to the content validity analysis.

### Intra Class Correlation and internal consistency reliability

The intra class correlation, ICC, was calculated on material from group 2. The time between test and retest in this study was between 36 and 367 days, with an average of 108 days. Cronbach’s alpha, i.e. the internal consistency reliability was calculated on the material from group 1.

### Effect size and standardized response mean

The effect size (ES) and standardized response mean (SRM) were calculated on the pre- and postoperative material from group 1 and for every individual health measurement instrument.

The ES is the difference between the preoperative and a postoperative score, divided by the preoperative standard deviation. The SRM is the difference between pre- and postoperative scores divided by the postoperative standard deviation.

### Minimal detectable change and minimal important change

For the calculation of Minimal Detectable Change (MDC) we used the Standard Error of Measurement (SEM) and the formula: MDC = 1.96 x SEM x square root of 2, and the Minimal Important Change (MIC) was calculated anchor-based for satisfied patients using the formula: MIC = 2,5 × SEM. Both were done according to the description by de Vet et al. [20].

The statistical analyses were calculated using SPSS version 22.

## Results

### Validity

#### Criterion validity

**Fig. 1:**
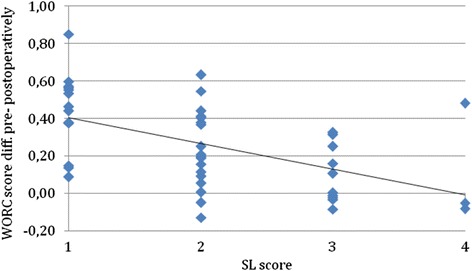
The SL vs. the difference between post- and preoperative WORC score. SL, the X-axis, ranges from 1 = very satisfied, to 4 = very unsatisfied. The Y-axis shows the difference between the pre- and postoperative result. Abbreviations: *WORC* Western Ontario Rotator Cuff index, *SL* Satisfactory level

Table 1 shows all the calculated inter-correlations between the different instruments. The analysis of how the patients’ SL responded to changes in WORC score pre- and postoperatively is shown in Fig. 1.

**Table 1:** The correlations between the individual health measurement instruments. Higher number means stronger correlation. A correlation of 1.0 is a complete correlation

	WORC	WOOS	OSS	EQ-5D
WOOS	0.97	-	-	-
OSS	0.74	0.73	-	-
EQ-5D	0.71	0.69	0.57	-
SL^a^	0.72	0.67	0.35	0.61
CS^b^	0.85	-	-	-

Abbreviations: *CS* Constant-Murley score, *WORC* Western Ontario Rotator Cuff index, *OSS* Oxford Shoulder Score. Pearson correlation coefficients except ^a^ Spearman’s correlation coefficient used for Satisfaction (SL), and only SL for postoperative study group 1 ^b^ CS correlation to WORC, material from study group 2

**Fig. 2:**
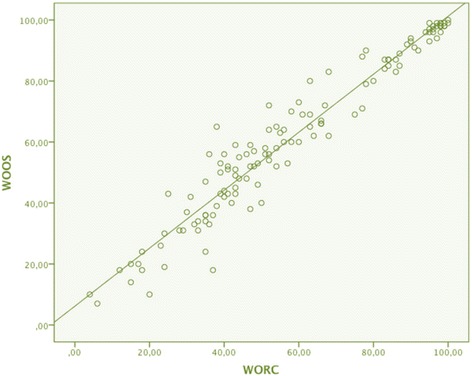
Scatter plot of WORC total scores vs. WOOS total scores. The results WORC and WOOS of 64 participants, both pre- and postoperatively (*N* = 128). PCC was 0.97 between WORC and WOOS. Abbreviations: *WORC* Western Ontario Rotator Cuff index, *WOOS* Western Ontario Osteoarthritis of the Shoulder index, *PCC* Pearson’s correlation coefficient

**Fig. 3:**
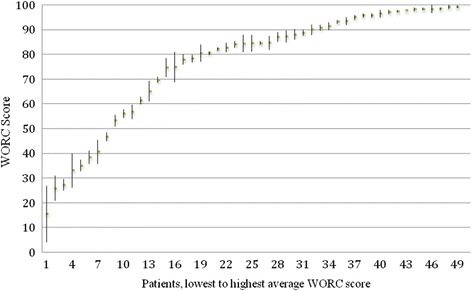
The test- retest material of WORC. The average of test and retest WORC score for every individual patient. The bars represent the range between test and the retest score

The PCC calculated between the WORC and WOOS was 0.97 (*p* < 0.001, *N* = 128). Figure 2 shows a scatter plot of WORC vs. WOOS scores to illustrate the correlation between the two scores. A strong correlation was revealed when comparing the 128 pairs of scores. In comparison the PCC calculated between the test and retest of the WORC was 0.97 (*p* < 0.001, *N* = 49), and a scatter plot of WORC test and retest scores is presented in Fig. 3.

#### Content validity

Floor and ceiling effects are shown in Table 2. These results showed that the specific health measurement instruments, WORC, WOOS, and OSS all had similar ceiling effects of approximately 10 % while the generic health instrument, EQ-5D, had a substantially higher ceiling effect.

**Table 2:** Floor and ceiling effects. Table showing the percentage of participants that was scoring minimum (floor) or maximum (ceiling) result

	Preop		Postop	
	Floor	Ceiling	Floor	Ceiling
WORC	0	0	0	7.7 %
WOOS	0	0	0	10.7 %
OSS	0	0	0	9.2 %
EQ-5D	0	0	0	32.3 %

Abbreviations: *WORC* Western Ontario Rotator Cuff index, *WOOS* Western Ontario Osteoarthritis of the Shoulder index, *EQ-5D* EuroQol – 5 dimensions, *OSS* Oxford Shoulder Scale

### Reliability

#### Intraclass correlation

The absolute average difference, i.e. disregarding whether a positive or negative change had occurred, between the WORC test and retest was 4 %.

For 8 % of the respondents, the final score differed by more than 10 % between the test and the retest (Fig. 3). The difference between the test and the retest scores was negative in 51 % of the cases, i.e. the second score was lower than the first, the scores were identical in 2 % (one case), and 47 % of the cases had a higher score in the retest. The ICC for the different domains ranged between 0.84 and 0.98 (Table 3).

**Table 3:** Reliability of WORC expressed as ICC

	ICC
WORC Total	0.97
Physical	0.92
Sport	0.92
Work	0.92
Lifestyle	0.98
Emotions	0.84

ICC calculated for WORC total scores as well as the individual domains of WORC

**Fig. 4:**
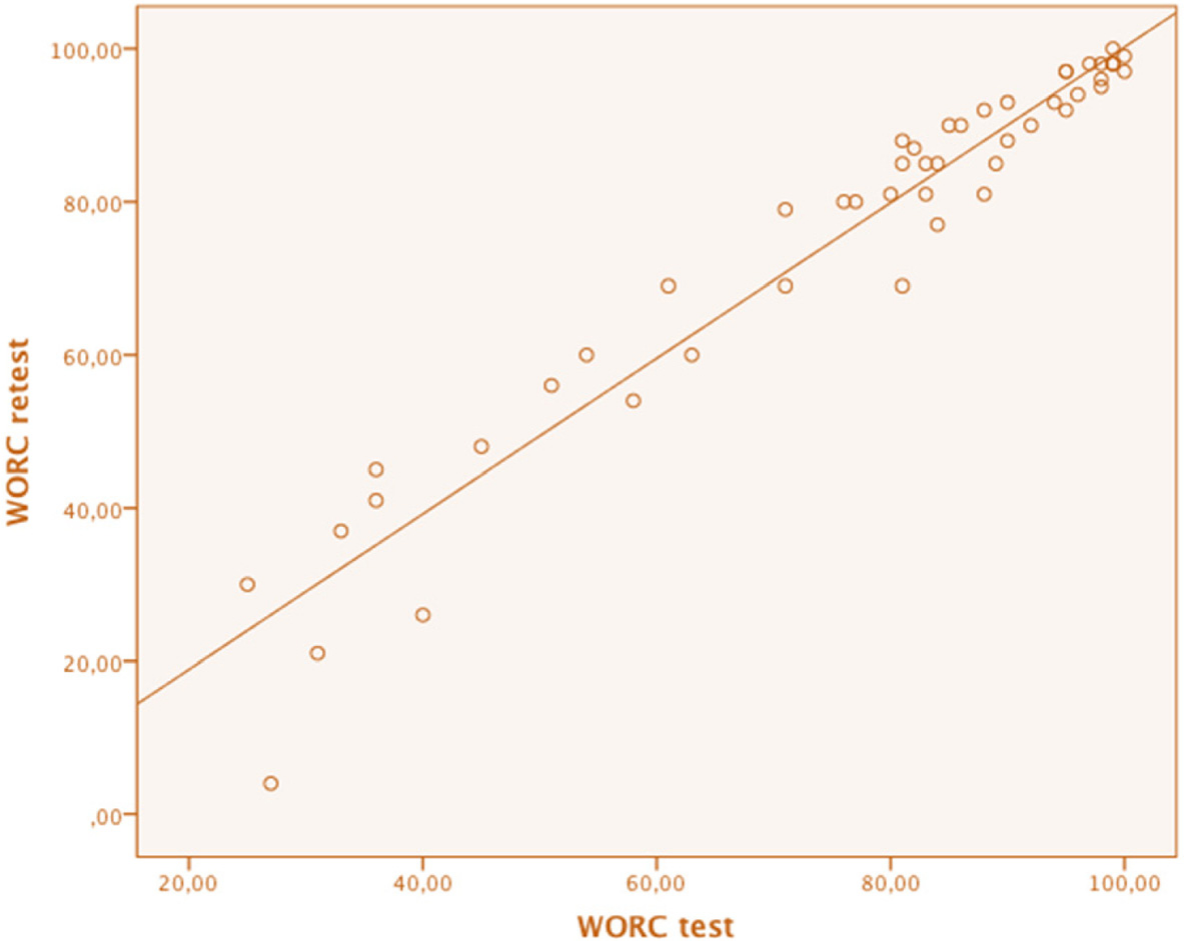
Scatter plot of WORC test vs. WORC retest. The individual results of the WORC test material plotted against the individual results of the WORC retest material (*N* = 49). Should be compared to Fig. 2

The calculated ICC of 0.97 (*p* < 0.001, *N* = 49) showed that there was no significant difference in the total result between the test and retest versions of the WORC, presented as a scatter plot in Fig. 4.

#### Internal consistency reliability

The result of the Cronbach’s alpha calculation suggested that the overall internal consistency reliability was high (0.97) when merging the pre- and postoperative material. The total score showed a higher Cronbach’s alpha than the individual domains, however the total score and the individual domains all showed high internal consistency and reliability (Table 4).

**Table 4:** Cronbach’s alpha of WORC. Cronbach’s alpha calculated for the total score and every domain, also pre- and postoperatively and combined (merging the preop. and postop. material)

	WORC	WORC	WORC
	preop	postp	combined
Total	0.93	0.98	0.97
Physical	0.83	0.92	0.90
Sport	0.69	0.91	0.89
Work	0.83	0.95	0.93
Lifestyle	0.83	0.92	0.91
Emotions	0.89	0.95	0.93

### Responsiveness

Responsiveness was tested for all instruments that had been answered both pre- and postoperatively. The WORC had a slightly higher ES than the WOOS (Table 5) but both showed excellent ES values (>0.80). The OSS also showed an excellent ES, although it was smaller than the ES values of the WORC and WOOS. The EQ-5D had the smallest effect size of 0.65 and was the only instrument to show a higher SRM than ES.

**Table 5:** Responsiveness results. Calculated ES and SRM

	ES	SRM
WORC	1,35	1.01
WOOS	1,28	1.05
OSS	0,95	0.67
EQ-5D	0,65	0.70

Abbreviations: *WORC* Western Ontario Rotator Cuff index, *WOOS* Western Ontario Osteoarthritis of the Shoulder index, *EQ-5D* EuroQol – 5 dimensions, *OSS* Oxford Shoulder Scale

According to our calculations within this study, both the MDC and MIC for the WORC were 10 %.

## Discussion

Our results suggest that the Swedish version of the WORC is indeed valid, reliable, and responsive enough to use in the evaluation of the QoL in patients with subacromial disease treated by surgery. We also found significant similarities between WORC and WOOS.

The criterion validity of 0.97 between the WORC and WOOS for assessment of subacromial disease can be considered to be strong. A possible explanation for this might be that the WORC and WOOS are structurally similar and are also constructed by the same researchers, using the same method [1]. The instruments have similar domains and number of items (21 for WORC and 19 for WOOS), and five of the items are identical. Previous studies have argued that a correlation between two health measurement instruments should be high enough to confirm a significant correlation, but if the correlation coefficient is close to 1.0, the additional value of adding the health measurements instrument in question will have very limited additional value [2].

The WOOS for patients with subacromial pain was psychometrically evaluated in a Swedish version by Klintberg et al. in 2012 [5]. In our study we have compared the WORC with several instruments of shoulder assessment, and with EQ-5D. The results of the correlation between the WORC, OSS and the CS confirm the view that the Swedish version of the WORC instrument can be considered to be valid for use on patients with subacromial pain. The CS has been used for correlation calculations in other studies that have reported moderate to high correlations (0.56–0.82) with WORC scores [1, 2, 11, 12, 21]. The results from the present study, however, show a higher correlation between the WORC and the CS (0.85) than do previous studies. In the study by Holtby, the correlation between the WORC and CS increased from 0.66 preoperatively to 0.82 postoperatively [21].

The WORC showed a strong correlation to the EQ-5D, indicating that subacromial pain actually has a substantial effect on the perception of general health. As the EQ-5D is a generic health instrument covering five dimensions of daily life, it could be expected that the EQ-5D would differ more in the correlation with the WORC than with shoulder specific instruments.

The criterion validity has been calculated in other studies for the translated versions of the WORC and our results are comparable to those of other studies when a generic health instrument was used as a criterion [2, 13, 15]. We used the EQ-5D as the generic health instrument while de Witte et al. used the SF-36 and calculated a PCC of 0.61 [2]. Due to the fact that the criterion validity can be considered to be high with respect to the WORC and the WOOS and the OSS and EQ-5D independent of each other, the interpretation is that the Swedish version of WORC indeed seems to reflect patient perception of subacromial pain.

The analysis suggests that the Swedish version of the WORC has firm content validity. There were neither floor nor ceiling effects preoperatively but all instruments had some ceiling effect postoperatively. The generic health instrument with fewer items (EQ-5D) had an unacceptably high ceiling effect of 32.3 % while the specific health instruments with a larger number of items (WORC, WOOS and OSS) had an acceptable ceiling effect of approximately 10 %. A probable explanation for this is that the fewer number of questions there are, the larger the proportion of answers that will end up at the scale limits, and EQ-5D is simply not a precise enough scale. A thorough investigation of the WORC psychometrics done by de Witte et al. in 2012 concluded similarly that an acceptable level of floor and ceiling effects was when less than 15 % of patients obtained minimum or maximum score, and de Witte found no floor or ceiling effects of the WORC [2].

The test-retest reliability of the WORC was strong (ICC = 0.97), and the separate domains also showed a high ICC, ranging from 0.84 to 0.98. In the original WORC study, an ICC of 0.95 was reported [1]. De Witte et al. [2] calculated an ICC of 0.89 and Kawabata et al. [15] calculated an ICC of 0.87 in the Japanese version of WORC. The result from the Klintberg study showed that the WOOS has strong reliability for evaluating subacromial pain, and that is similar to the reliability of the WORC seen in our study [5].

The results further show a Cronbach’s alpha for the WORC of 0.97. This is in line with previous translations and evaluations of the WORC, which had values ranging from 0.92 to 0.97 [2, 13, 15, 16]. It has been argued that a Cronbach’s alpha exceeding 0.95 might imply redundancy among the questions [16]. However, it is also a consequence of the statistical method that the more items there are, the higher the Cronbach’s alpha will be. Thus, the Cronbach’s alpha for the separate domains, due to fewer items, was slightly lower than for the total score and ranged from 0.89 to 0.93.

The questions in the sport domain seemed to be confusing for several participants. The domain includes questions regarding how the subacromial pain has affected the ability to do push-ups and to carry out throwing actions, both hard and far. Many participants never do these activities, which could possibly contribute to the lower internal consistency of the sport domain. However, when taking all of the other excellent reliability results into account, the poor preoperative reliability result of the sport domain does not alter the view that the Swedish version of the WORC is reliable.

Our results conclude that both the WORC and WOOS are responsive and have excellent capabilities to detect changes in subacromial pain. Previous studies of the WORC support the view from the current study that the WORC is a responsive instrument [2, 13]. The OSS showed a clear capability to detect changes in subacromial pain, but judging from the SRM the OSS was not as capable of detecting the same change as was the case for the WORC and the WOOS. A previous study by Ekeberg et al. showed a higher SRM for the OSS than in our study [17]. However, Ekeberg et al. calculated their results on a subgroup defined as improved by treatment, which could be an explanation for the higher SRM in their study. The EQ-5D has a lower responsiveness to changes in subacromial pain due to the fact that EQ-5D is a generic health instrument with few items.

There are some limitations in this study. The time interval in test-retest differed significantly between the patients, ranging from 36 to 367 days. Perhaps this is too long to ensure symptom stability. The optimal interval time for a test-retest has been a matter of some debate [22]. In previous studies, the time interval was often chosen with no clear reason given for the choice made [23]. However, the result of the test-retest analysis shows excellent reliability, indicating that the patient symptoms were actually in a stable phase at a minimum of one year from surgery.

This study has shown that the WORC is valid, reliable, and responsive. It also shows that it is possible to apply the WORC as a health measurement instrument for clinical use among patients with subacromial pain treated by surgery. However, any institution considering introducing the WORC as a health measurement instrument will have to consider how similar the WORC is to the WOOS. The suggestion from this study would be to choose either the WORC or WOOS since both are validated for use on patients with subacromial pain.

Further investigation of the usefulness of WORC and WOOS is certainly justified. The WORC might possibly have a higher validity or responsiveness among a working population than the WOOS since that domain is slightly more extensive in WORC. It may also be more likely the case that patients with subacromial and rotator cuff disease are to be found among working age patients than patients who suffer from arthritis.

## Conclusion

The Swedish-version of the WORC instrument can be considered reliable, valid, and responsive for use as a health measurement instrument on patients treated by surgery for rotator cuff syndrome and subacromial pain. The psychometric properties of the Swedish version of the WORC were in line with the original evaluation, as well as evaluations of different translations of the WORC. An additional finding was that the WORC and WOOS showed highly similar results in measuring outcome of surgical treatment for subacromial pain.

### Ethics and consent of participation

Approval by the Regional Ethical Review Board was obtained. (Dnr 2006/54-31/2). All the study subjects approved participation through writing their names on the self-evaluating functional scores used in the study.

### Consent to publish

Not applicable.

### Availability of data and materials

The de-identified data and material supporting the findings in this study could be provided through direct contact to the main author (SZ).

## Abbreviations

CS: Constant-Murley Score.
ES: Effect Size
ICC: Intraclass Correlation Coefficient
EQ-5D: European Quality of Life- 5 Dimensions 3 L
MCIC: Minimal Clinically Important Change
MIC: Minimal Important Change
MID: Minimally Important Difference
OSS: Oxford Shoulder Score
PCC: Pearson’s Correlation Coefficient
QoL: Quality of Life
RC: Rotator Cuff
SCC: Spearman Correlation Coefficient
SEM: Standard Error of Measurement
SF-36: Short Form- 36 item
SL: Satisfaction Level
SRQ: Shoulder Rating Questionnaire
SRM: Standardized Response Mean
WORC: Western Ontario Rotator Cuff Index
WOOS: Western Ontario Osteoarthritis of the Shoulder Index
